# Antibacterial effects and mechanisms of graphene oxide loaded on TiO_2_-nanotube-modified ti: an in vitro study

**DOI:** 10.1186/s12903-025-06453-w

**Published:** 2025-07-05

**Authors:** Keyi Wu, Xu Cao, Bin Luo, Caiyun Wang, Ran Lu, Xin Wang, Su Chen

**Affiliations:** 1 Laboratory of Biomaterials and Biomechanics, Beijing Key Laboratory of Tooth Regeneration and Function Reconstruction, Beijing Stomatological Hospital, Capital Medical University, Beijing, 100050 China; 2 Department of Dentistry, Radboud University Medical Center, Nijmegen, the Netherlands

**Keywords:** Titanium dioxide nanotubes, Graphene oxide, Porphyromonas gingivalis, Antibacterial effect

## Abstract

**Background:**

Peri-implant inflammation is an important factor affecting the success rate of dental implants. Improving the antibacterial performance of implants is an effective method to prevent and treat peri-implant inflammation.

**Methods:**

In this study, Graphene oxide (GO) was loaded onto Titanium (Ti) dioxide nanotubes (TNT) to produce a material with good antibacterial effects and biocompatibility. TNT was prepared through anodization, and GO-loaded TNT-modified Ti (TNT-GO) was synthesized through electrodeposition. The materials were characterized using scanning electron microscopy (SEM), Raman spectroscopy, X-ray diffraction (XRD), and atomic force microscopy (AFM). Standard plate counting assay, methyl thiazolyl tetrazolium (MTT) assay, and fluorescence staining were used to assess the antibacterial effects of the samples. Bacterial morphology observations were conducted to explore the antibacterial mechanisms via SEM, transmission electron microscopy (TEM), reactive oxygen species (ROS) assay, and lactate dehydrogenase (LDH) cytotoxicity assay. Cell proliferation and morphological observations of the materials were performed to test the biocompatibility of TNT-GO.

**Results:**

The characteristic peaks of graphene oxide on TNT-GO were detected by Raman spectra. The results of contact angle test and AFM show that TNT-GO had the greatest hydrophilicity and the largest surface roughness. When P. gingivalis was cultured on the sample surfaces, the TNT-GO demonstrated the lowest bacterial adhesion, with significantly disrupted bacterial morphology. Specifically, the bacterial cells exhibited collapsed and ruptured cell membranes, leakage of intracellular contents, reduced membrane density, and phospholipid extraction. The TNT-GO group also showed the highest release of LDH extracellularly and the greatest ROS generation. The results of CCK-8 show that there was no significant difference between TNT-GO group and Ti group. The results of adherent cells morphology observation show that human gingival fibroblasts on TNT-GO group were in fusiformis and irregular triangle shapes while human gingival fibroblasts on TNT group and Ti group were in round shapes.

**Conclusions:**

The TNT-GO had good biocompatibility and a good antibacterial effect on Porphyromonas gingivalis. It can inhibit bacteria-derived soft tissue infections and bone resorption and may be a very promising implant material.

## Introduction

Peri-implantitis is a bacteria-associated disease of soft tissue inflammation and progressive bone resorption around dental implants that can lead to implant loss [1, 2]. The pathogenic bacteria of peri-implantitis, such as Porphyromonas gingivalis (P. gingivalis), are similar to those of periodontitis [3]. However, soft tissue connections between natural teeth and dental implants are different. Dental implants have weak epithelial attachment and lack perpendicular connective tissue attachment, which makes dental implants susceptible to biofilm invasion and tissue destruction [4]. Therefore, it is essential to improve the antibacterial properties of the implants to prevent peri-implantitis. Titanium (Ti) and its alloys are the most extensively used implant materials because of their biocompatibility and excellent mechanical properties [5, 6]. However, more advanced titanium-based implants are needed because they remain vulnerable to biological inertia and infection [7]. Titanium dioxide nanotubes (TNT) synthesized by anodization are an implant surface modification method. Previous studies have shown that such TNT exhibited good biocompatibility and promoted the proliferation and adhesion of osteoblasts and fibroblasts, which is beneficial for osseointegration and soft tissue sealing [8–11]. As for the antibacterial property of TNT, studies have shown that it has lower amounts of adhered bacteria on its surface than Ti [12–14]. There are many ways to improve the antibacterial ability of TNT. For instance, their unique microstructure facilitates the incorporation of antibiotics [15–17], antimicrobial peptides [18, 19], and doping with antibacterial metals [20, 21] or non-metal elements [22, 23].

Graphene oxide (GO) is a graphene derivative and consists of functional groups such as hydroxyl, epoxy, carboxyl, and carbonyl. These functional groups enable GO to show enhanced solubility in aqueous solutions and potential binding sites for nanoparticles and proteins [24, 25]. Since the antibacterial effect of GO was first described in 2010 [26], much research about its antibacterial ability has been reported. According to studies, GO can enhance and inhibit bacteria growth depending on its concentration in solution. While GO can be a bacteria growth enhancer with low concentration, it can be a bacteria killer when its concentration is high [27–29]. The antibacterial effects of GO are not stable and are related to its concentration, lateral size, culture time, and type of bacteria exposure. To overcome these factors, GO-based composites can be fabricated by combining GO with other composites or non-GO nanomaterials. GO-based composites exhibit more stable and excellent antibacterial properties than pure GO [27, 30]. The antibacterial mechanisms of GO-based composites are less reported and need further research. The main antibacterial mechanisms of GO-based composites include (1) membrane disintegration mechanism; (2) oxidative stress mechanism; (3) phospholipid extraction mechanism [31]. However, most investigations of GO-based composite’s antibacterial mechanisms are based on experiments with bacteria such as E. coli and S. aureus. Studies about the antibacterial mechanisms of GO-based composites against peri-implantitis pathogenic bacteria such as P.gingivalis are rarely reported.

The biocompatibility of GO-based composites is considered controversial. The factors affecting the biocompatibility of GO-based composites include concentration, lateral dimensions, surface modification, and surface energy [31]. According to our previous study [9], a composite fabricated by loading GO onto TiO_2_-nanotube-modified Ti (TNT-GO) enhanced the proliferation and adhesion of human gingival fibroblasts (HGFs) at a GO doping concentration of 0.1 mg/mL. Previous research showed that TNT-GO with 0.1 mg/mL doping concentration of GO exhibited no apparent antibacterial effects. In this study, we increased the doping concentration of GO to 0.2 mg/mL. We explored whether TNT-GO had antibacterial properties against P. gingivalis and biocompatibility with HGFs when the doping concentration of GO was up to 0.2 mg/mL.

Overall, enhancing the antibacterial properties of dental implants is an effective strategy to reduce the incidence of peri-implantitis. However, existing methods for improving implant antibacterial performance still require further optimization. Based on our group’s previous research, this study employed an electrochemical deposition method to fabricate TNT-GO on Ti and investigated its antibacterial efficacy against P. gingivalis. The findings aim to provide an innovative approach for implant surface modification. The null hypothesis is that TNT-GO does not exhibit better antibacterial performance.

## Materials and methods

### Preparation of materials

Anodization was used to prepare TNT [32]. Before anodization, titanium foil (3.0 × 3.0 cm, 0.2 mm thickness, grade 4) and Platinum (Pt) foil (1.0 × 1.0 cm, 0.2 mm thickness) were ultrasonically cleaned in acetone, ethanol, and deionized water for 5 min sequentially, and then dried in air. Anodization was performed at a constant voltage of 50 V for 15 min in an electrolyte solution composed of 89.5 vol% ethylene glycol, 10 vol% deionized water, 0.5 vol% hydrofluoric acid, 0.5 wt% ammonium fluoride at room temperature. Ethylene glycol plays a role in regulating the viscosity of the electrolyte, slowing down the ion migration rate, and broadening the voltage window, which is conducive to the formation of long and ordered nanotubes. Hydrofluoric acid acts to rapidly dissolve the surface oxide layer of titanium, triggering the growth of nanotubes. Ammonium fluoride serves to provide a controllable source of fluoride ions (F⁻) and buffer the pH value, thereby influencing the diameter and wall thickness of the nanotubes. The electrolyte solution was magnetically stirred during anodization. The Ti foil as the anode and Pt foil as the cathode were kept in the electrolyte solution at a distance of 2 cm during anodization. After anodization, titanium dioxide nanotube arrays, which were an amorphous phase, were generated on the foil surface. The specimens were rinsed with ethanol and deionized water, and air-dried. Then the samples were annealed at 500 ◦C in the air for 2 h to attain a crystalline anatase structure, marked as TNT.

A GO solution (0.2 mg/mL) was obtained by diluting the original GO solution (2 mg/mL, 100681, XFNANO, Nanjing, China) with deionized water. The GO solution was sonicated for 2 h before electrodeposition. TNT-GO was synthesized via electrodeposition at a constant voltage of 50 V for 10 min. TNT was used as the anode, and Pt foil as the cathode. The two electrodes were placed 2 cm apart. During electrodeposition, the electrolyte was magnetically stirred at room temperature. After electrodeposition, the TNT-GO was rinsed with deionized water to remove residual unattached GO from the samples and dried in the air [33]. GO can enhance the physical properties of TNT, such as roughness and hydrophilicity. Additionally, it imparts the samples with the unique antibacterial properties of GO.

### Characterization of materials

Ti, TNT, and TNT-GO surface topographies were observed using field-emission scanning electron microscopy (SEM, S4800; Hitachi Ltd., Tokyo, Japan). X-ray diffraction (XRD; Ultima IV, Rigaku Co., Tokyo, Japan) examined the phase compositions of the samples. The distinct groups and bonds of Ti, TNT, and TNT-GO were analyzed using Raman spectroscopy (LabRAM HR800, HORIBA, Paris, France). The specific parameters used were the same as those used in a previous study [9]. The contact angle (CA) of specimens was detected by a contact angle analysis system (OCA15pro, Filderstadt, Germany). The surface roughness of the control and experimental samples (5 µm^2^) was measured using atomic force microscopy (AFM; Nanoscope V, Veeco Plainview, NY, USA).

### Antibacterial experiments

#### Bacteria culture

Porphyromonas gingivalis (P. gingivalis, ATCC33277) was cultured on tryptic soy agar (TSA) plates in an anaerobic environment (80% N_2_, 10% H_2_, 10% CO_2_) for 7 days to form bacterial colonies or cultured in brain heart infusion (BHI) solution under anaerobic conditions for 2 days to obtain a bacterial suspension. The culture temperature was 37 ◦C. The bacterial suspension was diluted to 10^8^ colony-forming units (CFU/mL) for follow-up experiments. For subsequent experiments, samples of Ti, TNT, and TNT-GO (1.0 × 1.0 cm) were sterilized by ultraviolet light (2 h).

#### Standard plate counting assay

Ti, TNT, and TNT-GO were incubated with 500 µL of P. gingivalis suspension (10^8^ CFU/mL) per well in the 24-well plates under anaerobic conditions at 37 ◦C for 24 h. After 24 h of incubation, the bacteria on the samples were collected and continuously diluted tenfold by BHI. One hundred microliters of each diluted bacterial suspension were added to a TSA plate. The TSA plates were cultured in an anaerobic environment at 37 ◦C for 7 days. After 7-day culture, the bacterial colonies on the plates were counted.

#### Methyl Thiazolyl tetrazolium (MTT) assay

Ti, TNT, and TNT-GO were incubated with 500 µL of P. gingivalis suspension (10^8^ CFU/mL) in the 24-well plates under anaerobic conditions at 37 ◦C for 24 h. Afterward, 100 µL of MTT (5 mg/mL, Solarbio, China) was added to each well and incubated for 2 h at 37 ◦C in the dark. Next, 200 µL of dimethyl sulfoxide (DMSO) was added to each well. A 100 µL suspension of the samples was transferred to 96-well plates. The optical density (OD) at 490 nm was measured using a microplate spectrophotometer (SpectraMax Paradigm, Molecular Devices, USA).

#### Fluorescence staining

Ti, TNT, and TNT-GO were incubated with 500 µL of bacterial suspension (10^8^ CFU/mL) for 24 h. The samples were gently rinsed with deionized water three times and then stained for 15 min in the dark at room temperature using LIVE/DEAD BacLight™ Bacterial Viability Kits (L7012). The stained samples were observed using fluorescence microscopy (OLYMPUS, Tokyo, Japan).

### Antibacterial mechanisms of TNT-GO

#### Morphology observation of Bacteria through SEM

Ti, TNT, and TNT-GO samples were cultured with 500 µL of P. gingivalis suspension (10^8^ CFU/mL) in the 24-well plates for 24 h in an anaerobic environment at 37 ◦C to form biofilms on the surface of samples. All the samples were gently washed by Phosphate Buffered Saline (PBS) 3 times, subsequently fixed with 2.5% glutaraldehyde at 4 ◦C for 2 h, and dehydrated using different volume ratios of ethanol (30%, 50%, 75%, 85%, 95%, 100%) for 15 min successively. After the samples were dried and sprayed with gold, the morphology of the bacteria on the samples was observed by SEM (S4800; Hitachi Ltd., Tokyo, Japan).

#### Lactate dehydrogenase (LDH) cytotoxicity assay

To examine the amount of LDH released from the bacteria, which reflects the degree of membrane damage, an LDH cytotoxicity assay kit (C0016, Beyotime, Shanghai, China) was used. Ti, TNT, and TNT-GO were incubated with the P. gingivalis suspension for 24 h under the same conditions as above. Bacterial suspensions from each group were collected and centrifuged at 7000 × g at 4 ◦C for 10 min. Supernatants (120 µL) mixed with 60 µL of LDH detection solution were added to a 96-well plate and incubated for 30 min at room temperature in the dark. OD at 490 nm was measured using a microplate spectrophotometer (SpectraMax Paradigm, Molecular Devices, USA).

#### Membrane observation of bacteria through transmission electron microscopy (TEM)

After samples were cultured with P. gingivalis under the same conditions as above, bacteria attached to the samples were collected in PBS and collected by centrifugation at 7000 × g at room temperature for 10 min. The sediment from each group was washed gently with PBS and then fixed with 2.5% glutaraldehyde at 4 ◦C for 24 h. They were gently rinsed with PBS, fixed with 1% aqueous OsO_4_ for 2 h, and then washed with PBS. Samples were successively dehydrated with ethanol (30%, 50%, 70%, and 80%) for 10 min, 95% ethanol for 15 min, 100% ethanol for 50 min, and then with ethylene oxide for 30 min. Samples were immersed in ethylene oxide/ epoxy resin (1: 1) for 2 h and then in epoxy resin for 3 h. Samples embedded in epoxy resin were kept at 45 ◦C for 12 h and then at 72 ◦C for 24 h. Thin slices (70 nm) of each group were placed on grids and stained with 4% uranyl acetate (1:1 acetone/water) and 0.2% lead citrate (water) for 1 min. The samples were air-dried and observed using a transmission electron microscope (TEM, JEM-1400, Japan).

#### Reactive oxygen species (ROS) assay

The ROS assay was used to detect intracellular ROS in P. gingivalis on the surface of the samples. P.gingivalis suspension(10^8^ CFU/mL) was cultured with Ti, TNT, and TNT-GO in 24-well plates for 24 h in an anaerobic environment at 37 ◦C. Samples were gently washed with PBS and stained with the ROS assay kit (S0033S, Beyotime, Shanghai, China) for 20 min in the dark. The cells were then gently rinsed with PBS. Fluorescent staining images were observed using a fluorescence microscope.

### Biocompatibility of TNT-GO

#### Cell culture

HGFs (CRL-2014, ATCC) were purchased from ATCC (Manassas, VA, USA) and grown in Minimum Essential Medium α supplemented with 10 vol% fetal bovine serum (FBS) and 1 vol% penicillin-streptomycin solution at 37 ◦C and 5% CO_2_. All the reagents were purchased from Thermo Fisher Scientific (Waltham, MA, USA). Passages 3–8 of HGFs were used in the experiment.

#### Cell proliferation

HGFs (2 × 10^4^/well) were cultured with Ti, TNT, and TNT-GO in 24-well plates for 1, 3, and 5 days at 37 ◦C and 5% CO_2_. The proliferation of HGFs on the samples was measured using a Cell Counting Kit-8 assay (CCK-8, C6005, New Cell & Molecular Biotech Co., Ltd, Suzhou, China). OD) at 450 nm was determined using a microplate spectrophotometer.

#### Cell morphology observation

Fluorescence staining was used to observe the morphology of HGFs on the surface of the samples. HGFs (2 × 10^4^/well) were cultured with Ti, TNT, and TNT-GO in 24-well plates for 4 h at 37 ◦C and 5% CO_2_. Samples containing adherent cells were fixed with 4% paraformaldehyde at room temperature for 15 min. The cells were then rinsed with PBS three times and permeabilized with 0.2% Triton X-100 at room temperature for 30 min. The samples were washed with PBS three times and then stained with TRITC Phalloidin (CA1610, Solarbio, Beijing, China) for 30 min in the dark. The samples were rinsed with PBS three times and stained with DAPI (ZLI-9557, ZSGB-BIO, Beijing, China). The morphology of HGFs on the samples was observed using fluorescence microscopy (OLYMPUS, Tokyo, Japan).

### Statistical analysis

All experiments were repeated three times. Data are expressed as means ± standard deviation (SD). All data were analyzed using one-way ANOVA (*P* < 0.05) to determine statistical differences. Statistical analysis was performed using SPSS software version 26.0.

## Results

### Material characterization

**Fig. 1:**
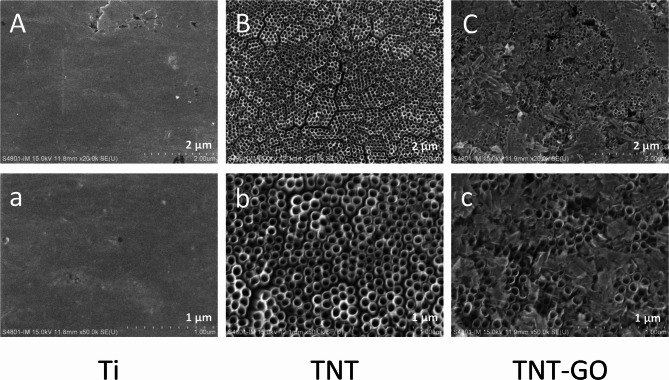
SEM images of Ti, TNT, TNT-GO. (**A**, **a**) The surface morphologies of Ti at different scales. (**B**, **b**) The surface morphologies of TNT at different scales. (**C**, **c**) The surface morphologies of TNT-GO at different scales

**Fig. 2:**
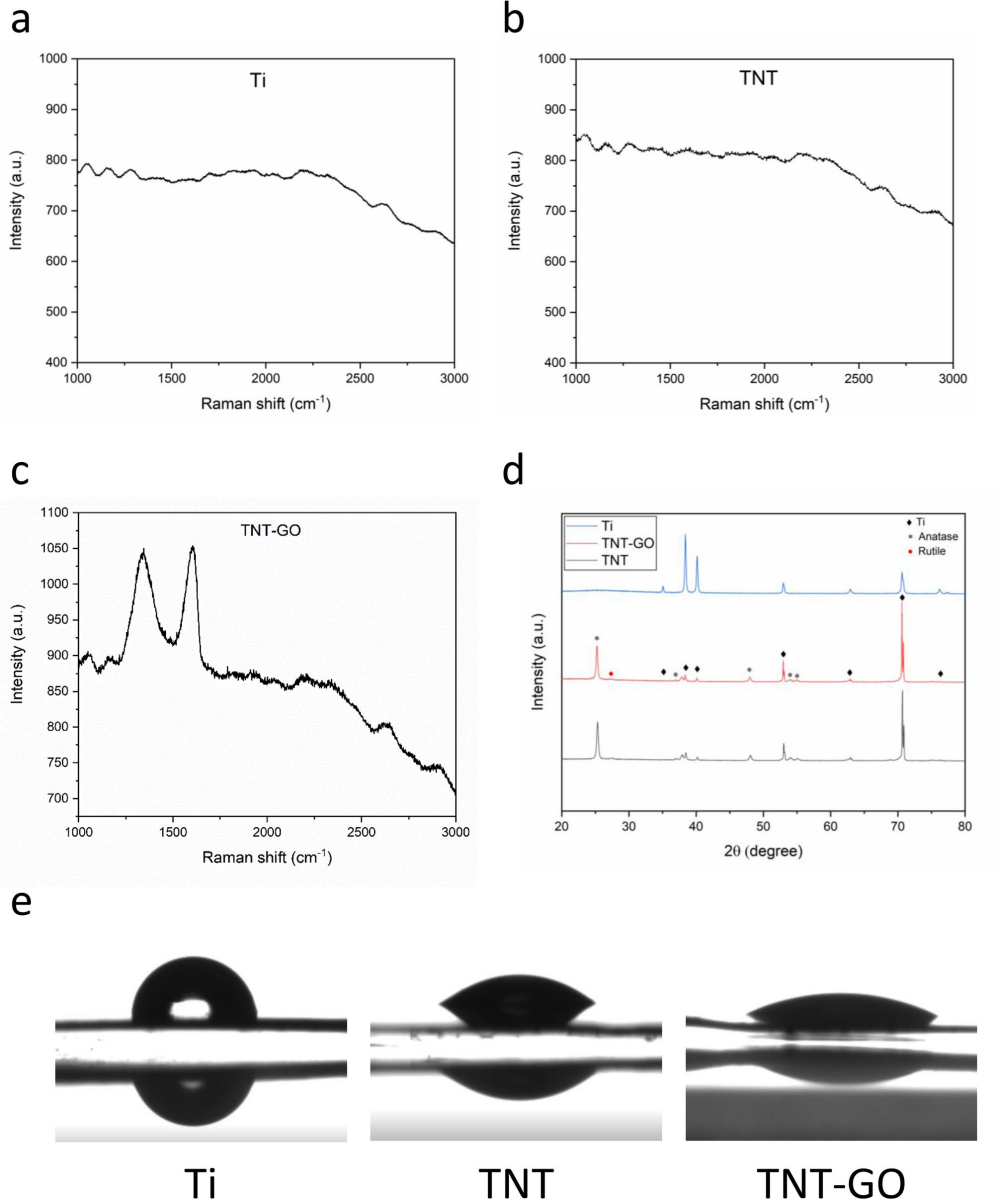
Characterization of Ti, TNT, TNT-GO. (**a**) Raman spectrum of Ti. (**b**) Raman spectrum of TNT. (**c**) Raman spectrum of TNT-GO. (d) XRD patterns. (**e**) Water contact angles of Ti, TNT, TNT-GO

The microstructures of Ti, TNT, and TNT-GO were observed using SEM (Fig. 1). As shown in Fig. 1A and a, the surface structure of Ti is smooth, with a few scratches. Figure 1B and b show that a nanotube array with an approximate diameter of 100 nm was fabricated on the surface of the TNT sample. The SEM images of TNT-GO indicate that the GO layer partially covered the surface of the nanotube array (Fig. 1C and c). Raman spectra are shown in Fig. 2a and b, and 2c. Compared to the images of Ti and TNT, the Raman spectrum of TNT-GO has two characteristic peaks of graphene: the D band at 1350 cm^− 1^ and the G band at 1580 cm^− 1^. This demonstrated that GO was modified on the surface of the TNT-GO samples. In Fig. 2d, X-ray diffraction (XRD) of TNT and TNT-GO show that apart from diffraction peaks of Ti, anatase diffraction peaks and rutile diffraction peaks of TiO_2_ were observed after anodization and annealing at 500 ◦C of Ti. However, only the Ti diffraction peaks were observed in the XRD spectrum of the Ti sample. Table 1; Fig. 2e show the water contact angle and surface roughness results. The contact angles of the samples decreased in the following order: Ti (88.2 ± 3.2°), TNT (49 ± 2.0°), and TNT-GO (32.2 ± 1.1°). TNT-GO had the most significant surface roughness, 50.4 ± 6.1 nm, while TNT was 30.5 ± 4.7 nm. Ti had the smallest surface roughness of 10.9 ± 2.6 nm. There was a statistically significant difference between the groups.

**Table 1:** Water contact angles and surface roughness of ti, TNT, TNT-GO

Sample	Contact Angle (°)	Surface Roughness (nm)
Ti	88.2 ± 3.2	10.9 ± 2.6
TNT	49 ± 2.0	30.5 ± 4.7
TNT-GO	32.2 ± 1.1	50.4 ± 6.1

Note: Data are expressed as means ± standard deviations (SD)

### Antibacterial effects

#### Standard plate counting assay

**Fig. 3:**
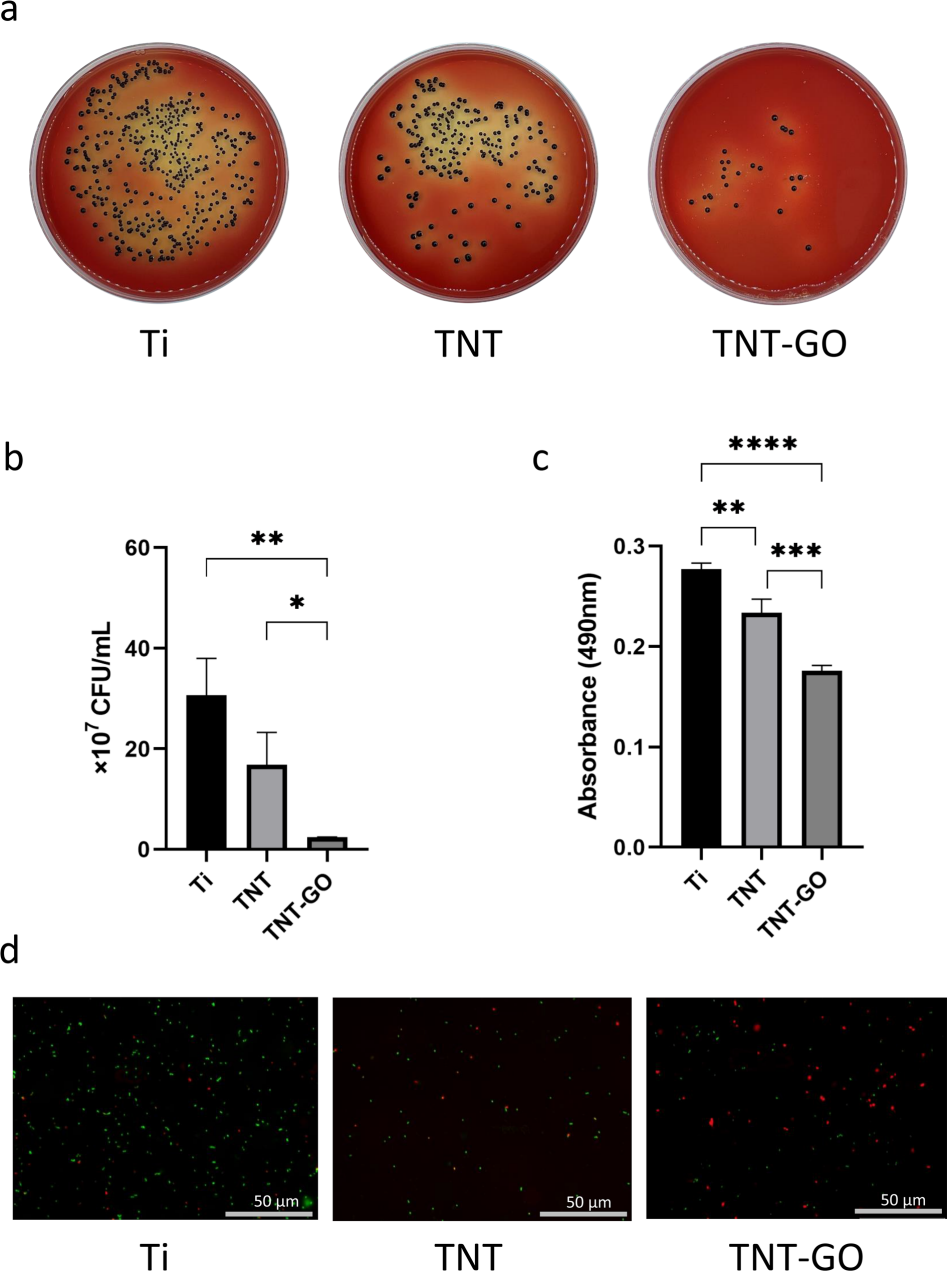
Results of antibacterial effects. (**a**, **b**) Photographs and quantitative analysis of standard plate counting assay. (**c**) MTT assay. (**d**) Images of LIVE/DEAD fluorescence staining on samples. (*P < 0.05. **P < 0.01. ***P < 0.001. ****P < 0.0001)

Figure 3a and b show the results of the standard plate counting assay. In Fig. 3a, the colony-forming units of the TNT-GO group were visually the least among the three groups, indicating a smaller number of P. gingivalis alive in TNT-GO group than the other two groups. Figure 3b shows the statistical quantitative analysis of colony-forming units among three groups. The number of colony-forming units in TNT-GO group was significantly lower than both TNT group (*P* < 0.05) and Ti group (*P* < 0.01). However, there was no significant difference in colony-forming units be-tween TNT group and Ti group (*P* > 0.05). These results reflect that TNT-GO showed the greatest antibacterial activity against P. gingivalis among the three groups, but the antibacterial activity of TNT was not improved compared to Ti group.

#### MTT assay

The results of the MTT assay are shown in Fig. 3c. The value of absorbance at 490 nm reflects the activity of bacteria. TNT-GO group had significantly lower value of absorbance at 490 nm compared to TNT group (*P* < 0.001) and Ti group (*P* < 0.0001), indicating that the bacteria in TNT-GO group had significantly lower activity than the other two groups. The absorbance of the TNT group was also significantly lower than that of the Ti group (*P* < 0.01). These results indicate that TNT-GO performed improved antibacterial properties against P. gingivalis than TNT and Ti groups.

#### Fluorescence staining

The LIVE/DEAD fluorescence staining was used to visually present the live/dead bacteria on the surface of three groups of samples. The results are shown in Fig. 3d. Green fluorescence indicates live bacteria, and red fluorescence indicates dead bacteria. The TNT-GO group had the smaller area of green fluorescence and the larger area of red fluorescence visually compared to TNT group and Ti group.

### Antibacterial mechanisms

#### SEM observation of bacteria morphology

**Fig. 4:**
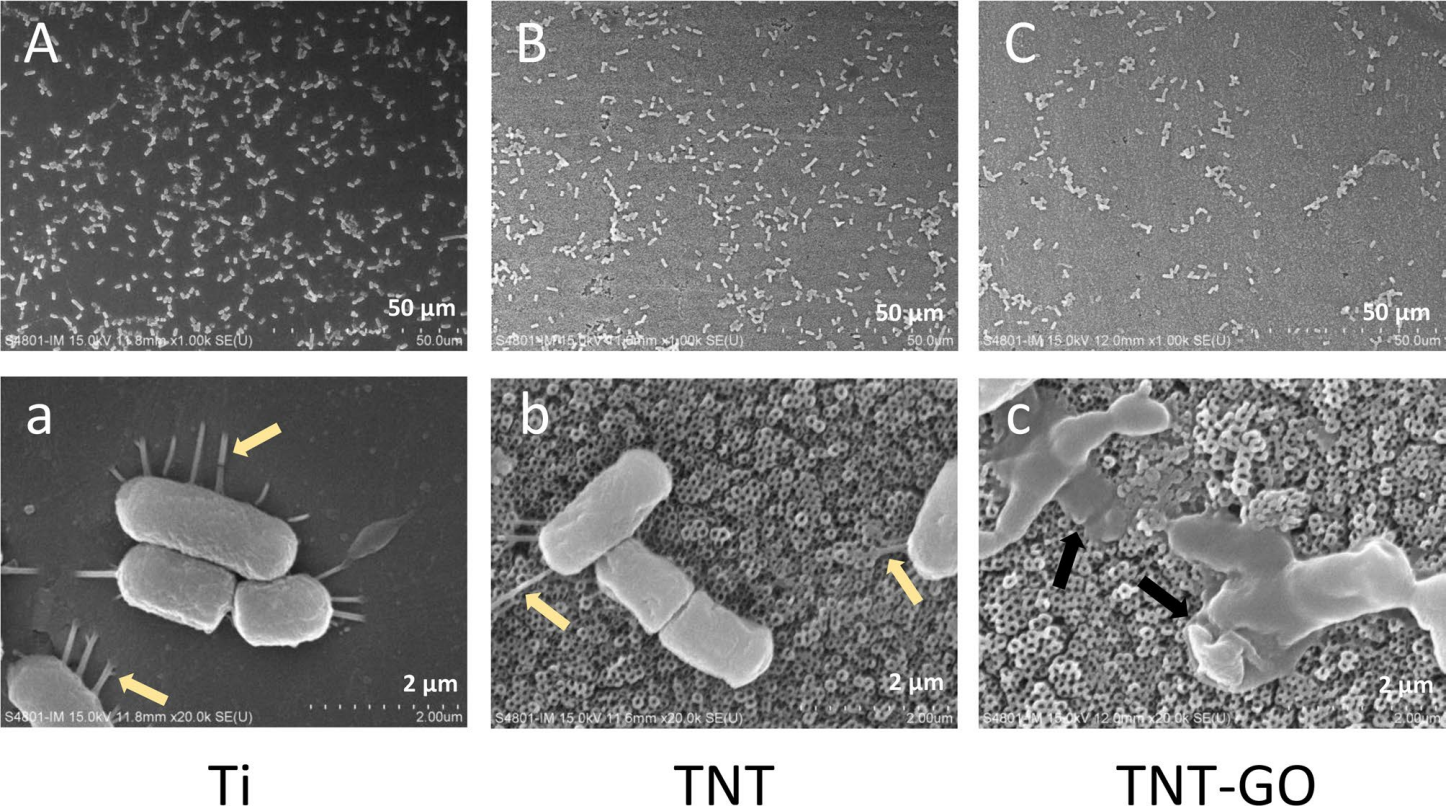
SEM images of P. gingivalis morphology on samples. (**A**, **a**) Ti. (**B**, **b**) TNT. (**C**, **c**) TNT-GO. Yellow arrows indicate bacteria pili. Black arrows indicate cell membrane damage

The morphology of P. gingivalis on the surface of samples observed by SEM is shown in Fig. 4. The density of P. gingivalis on samples were shown in Fig. 4A, B and C. The TNT-GO group had the smallest number of bacteria (Fig. 4C) compared with the Ti (Fig. 4A) and TNT groups (Fig. 4B). Bacteria in the Ti and TNT groups were plump in shape with pili extended (indicated by yellow arrows in Fig. 4a and b). However, bacteria in the TNT-GO group were irregular in shape and without pili, and showed various extents of membrane shrinkage and damage (indicated by the black arrows in Fig. 4c). The bacterial membrane morphology on TNT-GO suggests that the antibacterial property of TNT-GO was due to the membrane destruction of P. gingivalis when in contact with TNT-GO.

#### TEM observation of bacteria morphology

**Fig. 5:**
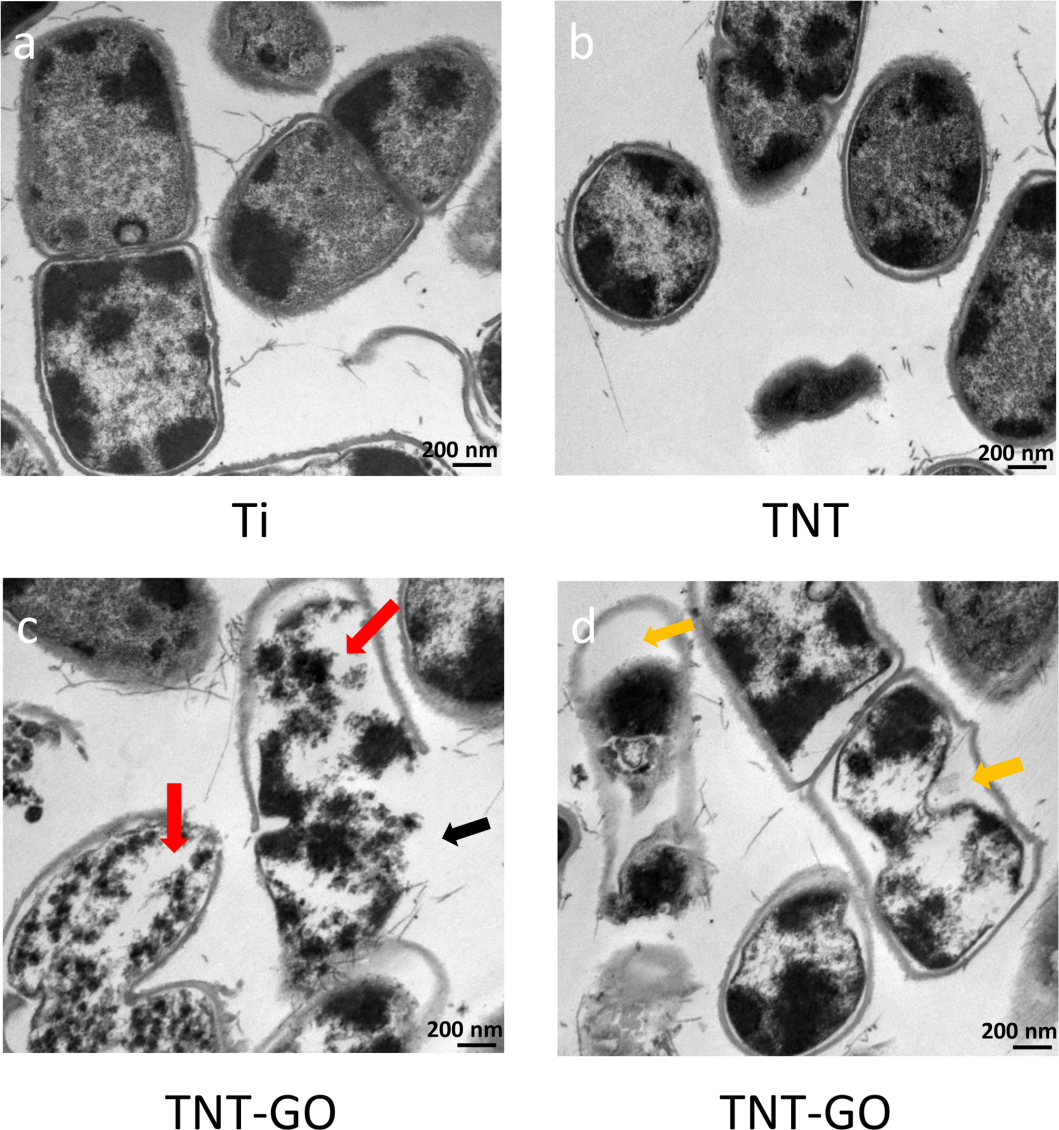
(**a**)TEM image of P. gingivalis morphology on Ti. (**b**) TEM image of P. gingivalis morphology on TNT. (**c**, **d**) TEM images of P. gingivalis morphology on TNT-GO. Red arrows indicate the reduced density of phospholipids. Black arrow indicates the membrane destruction and leakage of intracellular content. Yellow arrows show “nest empty,” which means membrane disintegration

To observe the membrane morphology of P. gingivalis on samples, TEM was used, and results were presented in Fig. 5. Figures 5a (Ti) and 5b (TNT) show the normal membrane morphology of P. gingivalis in the Ti and TNT groups, respectively. Figure 5c and d show the images of P. gingivalis on the surface of the TNT-GO group. In Fig. 5c, the black arrow indicates damage to the cell membrane and leakage of the intracellular content. The red arrows in Fig. 5c show the lower density of cell membranes in the TNT-GO group, indicating that phospholipids were extracted from the membrane. The image of P. gingivalis in Fig. 5d displays the disintegration of the cell membrane with an “empty nest” marked by yellow arrows, which indicates the loss of cytoplasm and membrane destruction.

#### LDH cytotoxicity assay

**Fig. 6:**
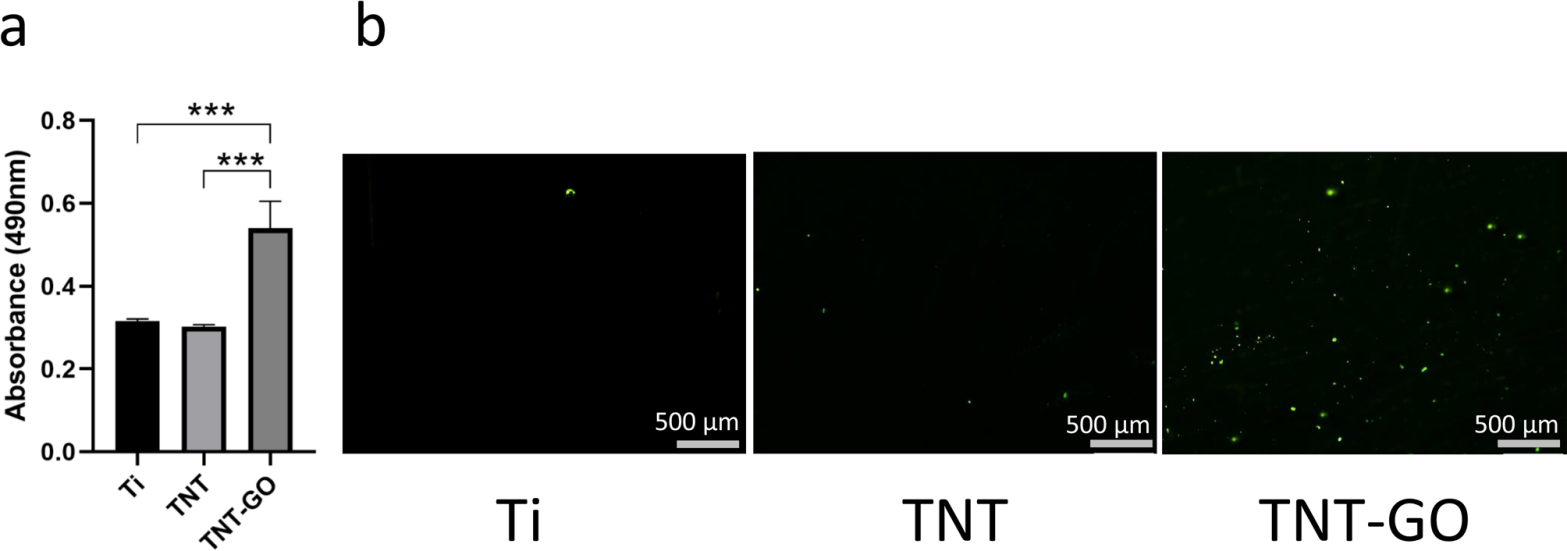
(**a**) LDH cytotoxicity assay. (**b**) ROS fluorescence staining images of Ti, TNT, TNT-GO. (***P < 0.001)

Figure 6a shows the results of the LDH cytotoxicity assay, which reflects the degree of bacterial membrane damage. The higher value of absorbance at 490 nm indicates the larger amount of lactate dehydrogenase (LDH) and higher degree of bacterial membrane damage. The absorbance value of the TNT-GO group was significantly higher than both TNT group (*P* < 0.001) and Ti group (Ti) (*P* < 0.001). However, there was no statistically significant difference between the Ti and TNT groups. These results indicate that membrane disintegration played a role in the antibacterial activity of TNT-GO against P. gingivalis.

#### ROS assay

To explore whether ROS was produced by P. gingivalis, ROS assay was conducted. The results of the ROS assay are shown in Fig. 6b. Green fluorescence indicates ROS produced by P. gingivalis. TNT-GO exhibited the largest green fluorescence area. However, ROS were rarely observed in the Ti and TNT groups. These results indicate that the level of oxidative stress inside P. gingivalis in the TNT-GO group was high, which could lead to bacterial death on the surface of TNT-GO.

### Biocompatibility

#### Cell proliferation

**Fig. 7:**
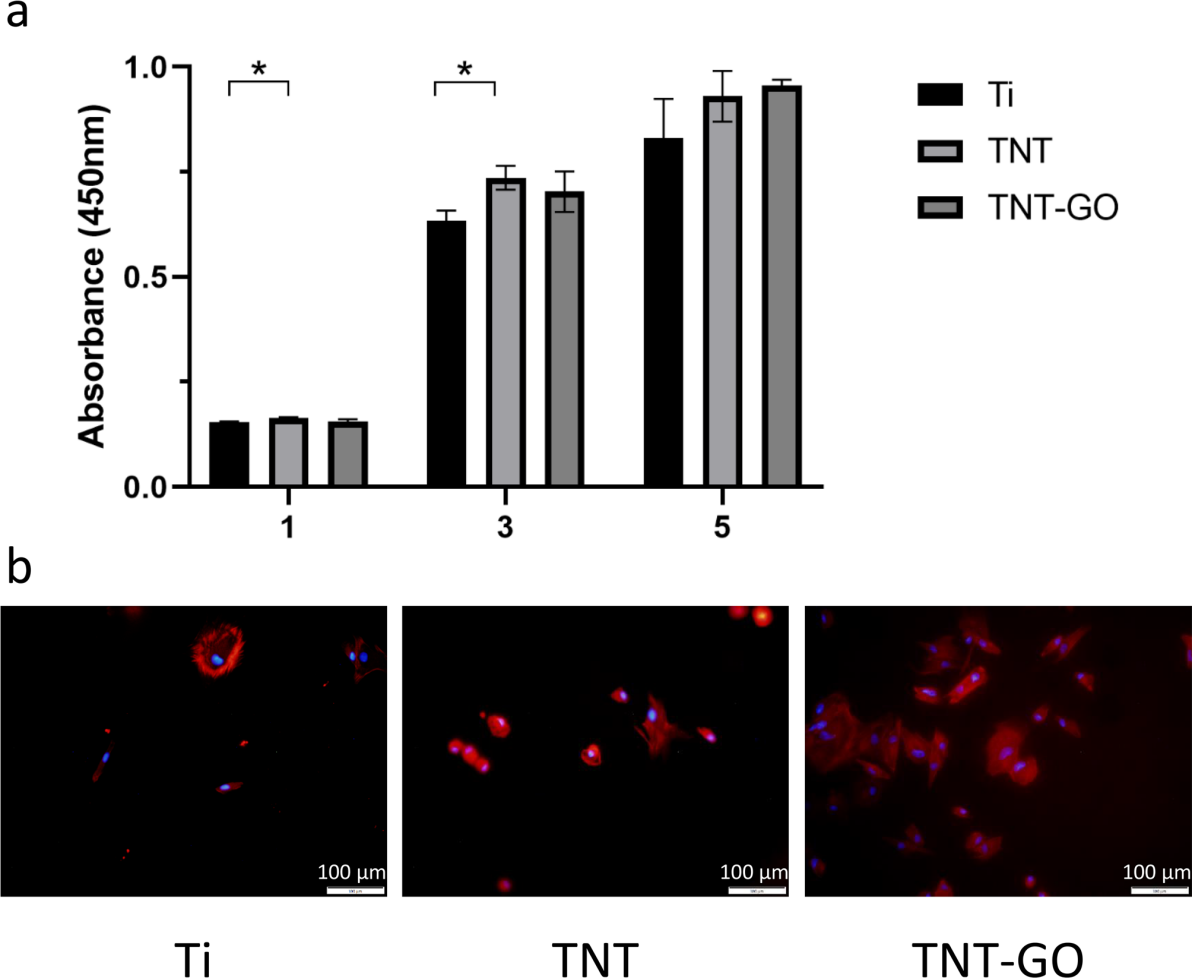
(**a**) Cell proliferation of HGFs on Ti, TNT, TNT-GO for 1, 3, 5 d. (**b**) Fluorescence staining images of HGFs on samples at 4 h. (*P < 0.05)

The results of HGFs proliferation on the surface of the samples after 1, 3, and 5 days tested by CCK-8 are shown in Fig. 7a. The proliferation of HGFs in the TNT-GO group was not significantly different from that in the Ti group by comparing the absorbance value at 450 nm between the two groups (*P* > 0.05). The TNT group showed significantly enhanced proliferation of HGFs on days 1 and 3 compared to Ti group (*P* < 0.05), which is consistent with the previous study of our research team [9].

#### Cell morphology

Figure 7b shows the morphology of adherent HFGs on Ti, TNT, and TNT-GO after 4 h. Blue fluorescence indicates the nucleus, and red fluorescence indicates the F-actin-labeled cytoskeleton. The HGFs in the Ti and TNT groups were round in shape. The HGFs on TNT-GO spread out in fusiform and irregular triangular shapes. The morphology of HGFs demonstrated that TNT-GO promoted HGFs adhesion.

## Discussion

In this study, we synthesized TNT-GO via anodization and electrodeposition, explored its antibacterial properties and mechanisms of action against P. gingivalis, and evaluated its biocompatibility with HGFs in vitro. TNT-GO was prepared by electrodeposition in a suspension of GO with molecular diameters ranging from 50 nm to 200 nm. The small size of GO results in its good dispersion in aqueous solution and partial attachment to the surface of TNT-GO. The results of TNT-GO characterization by SEM, Raman spectroscopy, XRD, AFM, and water contact angle tests revealed that TNT-GO had characteristic topography and structure. These features of TNT-GO resulted in antibacterial effects and biocompatibility.

As shown in Fig. 3a and d, and 4, TNT-GO exhibited antibacterial properties by decreasing bacterial adhesion on its surface, which could be due to charge repulsion, surface roughness, and wettability [8]. Many studies have demonstrated that electrostatic interactions between differently charged surfaces of bacteria and materials affect bacterial adhesion [34, 35]. TNT-GO surfaces present negative charges owing to the terminal hydroxyl groups of TNT and ionization of the carboxylic groups of GO [8, 36]. The outer layer of gram-negative bacteria such as P. gingivalis consists of lipopolysaccharide (LPS), which results in a negatively charged surface [37]. The charge repulsion produced by the same charges between P. gingivalis and TNT decreased the initial adhesion of bacteria and led to the antibacterial effect of TNT. Moreover, the surface roughness and wettability could have played a role in preventing bacterial adhesion. Generally, it is widely accepted that an increase in surface roughness leads to enhanced bacterial adhesion. However, when the roughness is at the nanoscale, bacterial adhesion decreases with increasing surface roughness [38], which has been observed in several studies [39, 40]. Liu et al. also found the increased nanoscale surface roughness of polydimethylsiloxane alone can inhibit both Gram-positive and Gram-negative bacteria adhesion for up to 2 days [41]. They detected the possible mechanisms that the increased nanoscale surface roughness and associated energy may influence select casein adsorption and then lead to the decrease of bacteria absorption. The hydrophilicity of materials may influence bacteria adhesion as well. It was observed that bacterial attachment is more prone to hydrophobic surfaces than hydrophilic surfaces [42, 43]. This phenomenon may be explained by the fact that a hydrophilic surface attracts water and can lead to water film formation. The adhesion of bacteria with hydrophobic surfaces on a hydrophilic surface would be lower due to the process to remove the adsorbed water before adhesion takes place when compared with bacteria adhesion on a hydrophobic surface [44]. Table 1; Fig. 2e show that TNT-GO had the most considerable surface roughness and the best hydrophilicity, which can lead to the lowest adhesion of P. gingivalis in the TNT-GO group compared to the other two groups. Hence, the decreased bacterial attachment on TNT-GO can be explained by its negatively charged surface, high surface roughness, and hydrophilicity.

**Fig. 8:**
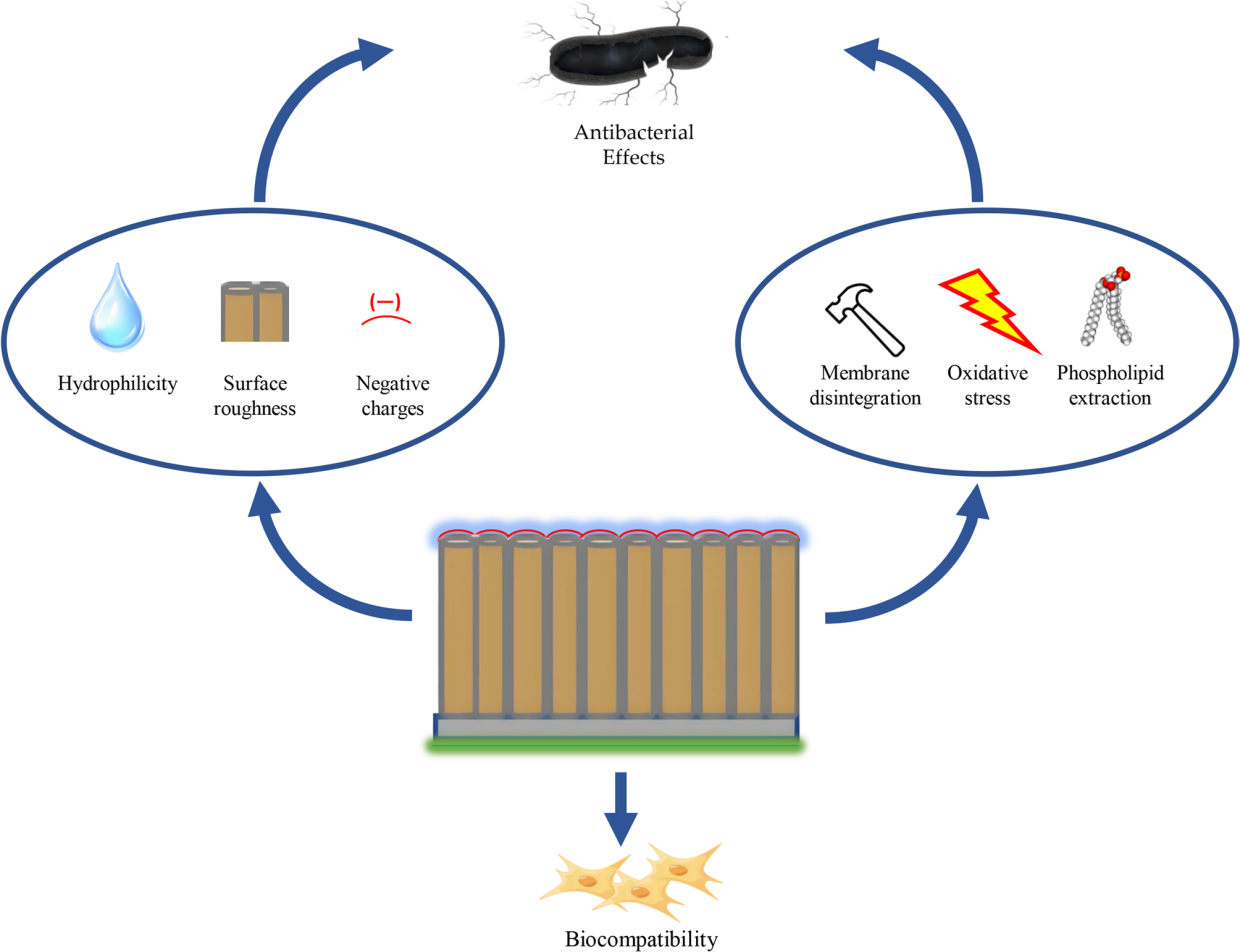
Schematic representations of antibacterial activity for TNT-GO

In addition, GO plays a crucial role in bacteria’s growth inhibition and death. However, the antibacterial mechanisms of GO still need to be understood entirely. In this study, we explored three possible mechanisms (Fig. 8). The first is membrane disintegration. The sharp edges of GO lead to bacterial death through contact with bacteria, disrupting membrane structure and leakage of intracellular content [26, 27, 29, 45, 46]. The SEM images of bacterial morphology observed through SEM presented an irregular shape and damaged cell membrane of P. gingivalis on the surface of TNT-GO (Fig. 4c). Through TEM, the membrane disintegration of P. gingivalis in the TNT-GO group is indicated by black and yellow arrows (Fig. 5c and d). The leakage of intracellular substances, such as LDH, reflects the degree of membrane destruction [47]. As shown in Fig. 6a, the highest amount of LDH in the TNT-GO group demonstrated the highest degree of membrane damage. Membrane disintegration of P. gingivalis on TNT-GO can be caused by the sharp edges of GO on the cell membrane, resulting in bacterial death.

Oxidative stress is another widely accepted mechanism. Intracellular oxidative stress causes bacterial inactivation and death by disrupting bacterial metabolism and essential bacterial functions [48]. Reactive oxygen species (ROS) such as superoxide anions (O_2_^•−^), hydrogen peroxide (H_2_O_2_), singlet molecular oxygen (^1^O_2_), and hydroxyl radicals (OH•) are representative of the level of oxidative stress [45, 48]. ROS can cause lipid peroxidation of membranes, oxidative damage to DNA, and irreversible destruction of individual amino acids and peptide backbones in proteins [31, 49, 50]. This study used the ROS assay to detect ROS levels in P. gingivalis cultured with Ti, TNT, and TNT-GO. The fluorescence staining images (Fig. 6b) indicated that the TNT-GO group had the highest level of ROS, which can result in the death of P. gingivalis. Associated with the results of LIVE/DEAD fluorescence staining (Fig. 3d), dead P. gingivalis on the surface of TNT-GO may be related to the high level of intracellular ROS.

The phospholipid extraction mechanism is lesser known compared with the first two mechanisms. There is an interaction between GO and lipids in the bacterial cell membrane, which is stronger than the attraction between lipids and the inner cell membrane, owing to the specific two-dimensional structure with sp^2^ carbons of GO. After contact with GO, lipid molecules are extracted from the inner and outer membranes, leading to membrane collapse and bacteria death [47, 51]. The bacterial morphology observed by TEM (Fig. 5c) demonstrates that GO-extracted lipids from the membrane (indicated by red arrows) played a role in bacterial membrane destruction and inactivation.

However, the cytotoxicity and biocompatibility of GO are complex. Some studies have shown that GO promotes stem cell differentiation, proliferation, and expansion [28, 52], which are associated with numerous factors, such as concentration, lateral size, dispersion, and surface modification of GO [31, 53]. In a previous study, we discovered that TNT-GO promoted the proliferation and adhesion of HGFs when the concentration of GO suspension for electrodeposition was 0.1 mg/mL [9]. This phenomenon can be explained by the nanostructure, surface roughness, enhanced hydrophilicity, and protein adsorption of TNT-GO [50, 54, 55]. In this study, we synthesized TNT-GO with a higher concentration of the GO suspension (0.2 mg/mL) to obtain a better antibacterial effect. The adhesion of HGFs in the TNT-GO group was still the best among the three groups (Fig. 7b), which demonstrated that the increasing GO amount from 0.1 mg/mL to 0.2 mg/mL in electrodeposition did not affect HGFs adhesion. The proliferation of HGFs on TNT-GO with a high amount of GO showed no statistical difference from that of the control group (Ti) (Fig. 7a). This may be due to the higher quantity of GO attached to the surface of TNT-GO than in the previous study.

Our study explored the antibacterial properties and possible mechanisms of TNT-GO against P. gingivalis and demonstrated that TNT-GO has good biocompatibility with HGFs. However, this study has certain limitations. Firstly, only a single concentration of GO (0.2 mg/mL) was investigated, and additional concentration gradients should be tested in future studies to better define the optimal antibacterial dose. Secondly, the antibacterial effects of TNT-GO were evaluated exclusively in vitro, and further in vivo experiments are required to validate its functional efficacy and biosafety.​​ In future work, we plan to conduct more comprehensive in vivo studies to systematically assess the antibacterial performance and safety profile of TNT-GO. Additionally, we will establish multiple concentration groups to identify the optimal concentration that maximizes antibacterial efficacy while minimizing cytotoxicity. These efforts aim to provide innovative strategies for the surface modification of dental implants and abutments, ultimately improving their clinical performance and long-term stability.

## Conclusions

In this study, TNT-GO was fabricated via anodization and electrodeposition. Furthermore, the TNT-GO produced herein exhibited antibacterial effects against P. gingivalis, which can be attributed to the material characteristics of TNT-GO, such as charge repulsion, surface roughness, hydrophilicity, and the antibacterial effects of GO through membrane disintegration, intracellular oxidative stress, and phospholipid extraction from the cell membrane. TNT-GO showed no cytotoxicity and exhibited good biocompatibility with the HGFs. However, the in vivo antibacterial effects require further investigation. TNT-GO may be a promising dental implant material with good antibacterial effects against P. gingivalis, which can inhibit bacteriogenic soft tissue infections and bone resorption.

## Data Availability

All data generated or analyzed during this study are included in this published article.

## Abbreviations

Ti: Titanium
TNT: Titanium dioxide nanotubes
TNT-GO: GO-loaded TNT-modified Ti
SEM: Scanning electron microscopy
XRD: X-ray diffraction
AFM: Atomic force microscopy
TEM: Transmission electron microscopy
ROS: Reactive oxygen species
LDH: Lactate dehydrogenase
P. gingivalis: Porphyromonas gingivalis
GO: Graphene oxide
HGFs: Human gingival fibroblasts
CA: Contact angle
TSA: Tryptic soy agar
BHI: Brain heart infusion
DMSO: Dimethyl sulfoxide
OD: Optical density
FBS: Fetal bovine serum
LPS: Lipopolysaccharide
